# Well-Being After Severe Brain Injury: What Counts as Good Recovery?

**DOI:** 10.1017/S0963180121000086

**Published:** 2021-10

**Authors:** Mackenzie Graham, Lorina Naci

**Affiliations:** 1 Wellcome Centre for Ethics and Humanities, Big Data Institute, University of Oxford, Oxford, UK; 2 School of Psychology and Global Brain Health Institute, Trinity College Dublin, Dublin, Ireland

**Keywords:** disorders of consciousness, brain injury, well-being, decisionmaking, recovery disorders of consciousness

## Abstract

Disorders of consciousness (DOC) continue to profoundly challenge both families and medical professionals. Once a brain-injured patient has been stabilized, questions turn to the prospect of recovery. However, what “recovery” means in the context of patients with prolonged DOC is not always clear. Failure to recognize potential differences of interpretation—and the assumptions about the relationship between health and well-being that underlie these differences—can inhibit communication between surrogate decisionmakers and a patient’s clinical team, and make it difficult to establish the goals of care. The authors examine the relationship between health and well-being as it pertains to patients with prolonged DOC. They argue that changes in awareness or other function should not be equated to changes in well-being, in the absence of a clear understanding of the constituents of well-being for that particular patient. The authors further maintain that a comprehensive conception of recovery for patients with prolonged DOC should incorporate aspects of both experienced well-being and evaluative well-being.

## Introduction

The past several decades have marked significant advances in our understanding of disorders of consciousness (DOC). Research clarifying the underlying mechanisms of these disorders, combined with new methods of diagnosis and therapeutic approaches, have brought new hope of meaningful recovery for a proportion of patients.

Despite these advances, DOC continue to profoundly challenge both families and medical professionals. Diagnosis at the bedside remains tortuous and prognosis is often uncertain, particularly in the days and weeks after injury when many significant treatment decisions are made. Families and surrogate decisionmakers may find it difficult to understand the nature of the patient’s condition. Moreover, the clinical assessment of these patients is particularly difficult because of its reliance on subjective interpretation of inconsistent behaviors, which are often limited by motor constraints.

Once a brain-injured patient has been stabilized, questions turn to the prospect of recovery. What does “recovery” mean in the context of patients with prolonged DOC? On the one hand, recovery may be understood in terms of the recovery of function,^1^ ^,^^2^ ^,^^3^ ^,^^4^ a perspective broadly—albeit not exclusively—adopted by physicians.^5^ On the other hand, families often adopt a more general perspective, understanding the patient’s injury in the context of the person that they were, and are, and understanding recovery in terms of a life that the patient would find worthwhile. These two perspectives are sometimes at odds with each other, and failure to recognize these differences can inhibit communication between surrogate decisionmakers and a patient’s clinical team, and impede the goals of care.

How we understand the idea of recovery depends on various assumptions about the relationship between health and well-being. In this paper, we examine this relationship as it pertains to patients with prolonged DOC. We argue that changes in awareness or other function should not be equated to changes in well-being, in the absence of a clear understanding of the constituents of well-being for that particular patient. Further, we maintain that a comprehensive conception of recovery for patients with prolonged DOC should incorporate aspects of both experienced well-being, and evaluative well-being.

## Disorders of Consciousness

DOC encompass a spectrum of syndromes, characterized by alterations in arousal and awareness, and classified according to the presence or absence of predefined behavioral markers. Coma is defined as the complete absence of arousal and awareness, the vegetative state (renamed “unresponsive wakefulness syndrome” [VS/UWS]) is defined as arousal without awareness, and the minimally conscious state (MCS) is defined as minimal, reproducible, but inconsistent evidence of awareness.^6^ Recently, MCS has been further categorized as MCS without language (MCS−), and MCS with language (MCS+); any of command-following, intelligible verbalization, or intentional communication are sufficient to indicate that a patient is MCS+.^7^

A recent addition to these diagnostic categories is “cognitive motor dissociation” (CMD), which is characterized by evidence of volitional brain activity detected by task-based neuroimaging, in patients who show complete absence of behavioral responsivity and whose bedside behavioral diagnosis suggests coma or VS/UWS.^8^

For both VS/UWS and MCS, the likelihood of significant functional recovery diminishes over time, with the cause of injury a strong determinant of outcome in both cases. Patients with nontraumatic brain injuries (e.g., anoxic) tend to have a shorter window for recovery, and more severe long-term disability, than patients with traumatic brain injury (TBI). Historically, the vegetative state has been defined as “persistent”/“prolonged” after 4 weeks postinjury, and “permanent” after 12 months postinjury for traumatic brain injury, and 6 months for nontraumatic brain injury (3 months in the United States). However, recent studies have shown that a substantial minority of patients will recover consciousness beyond this timeframe.^9^ ^,^^10^ ^,^^11^ This has prompted a shift in practice guidelines, as well as replacement of the term “permanent VS” with “chronic VS.”^12^

Accurate diagnosis of VS/UWS or MCS is challenging,^13^ yet critical to ensuring appropriate clinical decisionmaking and treatment planning, given that MCS is associated with a greater probability of significant functional recovery than VS/UWS.^14^ Judgements about patient prognosis after severe brain injury tend to rely heavily on clinical findings that can be obtained within the first 7 days postinjury,^15^ which typically include demographic information, markers of clinical severity (e.g., Glasgow Coma Scale score and pupillary reactivity), secondary insults (hypoxia and hypotension), vital signs, and results of computed tomography scans. Although prognostic models based on these factors, such as the International Mission for Prognosis and Analysis of Clinical Trials in TBI (IMPACT), have been developed as an early predictor of outcome for this patient group,^16^ they may not be sufficiently accurate to be the exclusive basis of decisions to limit life-sustaining treatment.^17^

Consequently, early outcome prediction rests largely on subjective clinical judgement. Yet, there is evidence that this may lead to an underestimation of the potential for recovery, and a corresponding failure to pursue aggressive treatment.^18^ For example, a retrospective study across six Canadian Level 1 trauma centers, involving 720 adult patients diagnosed with traumatic brain injury, found that 70% of patient deaths in the intensive care unit were related to withdrawal of life-sustaining therapy. In 65% of those patients, withdrawal of treatment occurred within 72 hours of admission.^19^ Behavioral or other clinical evidence indicative of future recovery may not emerge within this narrow timeframe, suggesting that a proportion of these patients may have been withdrawn from treatment prematurely.^20^

Conversely, other factors may push in the opposite direction, leading to continued treatment for patients with prolonged DOC, regardless of whether this is in their best interests. Research suggests that for patients with prolonged DOC, it is often families rather than clinicians that appeal for the withdrawal of life-prolonging treatment such as artificial nutrition and hydration.^21^ If families lack the pertinent clinical or legal knowledge, and this information is not conveyed to them by the clinical team, treatment will often continue by default. Clinicians may have ethical or religious objections which shape their communication with families about treatment withdrawal, or may themselves lack adequate knowledge or training about the complexities of prolonged DOC.^22^ ^,^^23^

## The Value of Health

Traditionally, the value of health has been understood in terms of its contribution to well-being, (i.e., how well a person’s life is going, for them). Health matters insofar as it is good or bad for people, and it is good or bad for people based on the ways it influences and contributes to their well-being.^24^ Accordingly, treatment of illness or injury is justified by the improvements to our well-being that it brings about.

On closer examination, however, we can see that the relationship between health and well-being is not quite so straightforward. Whereas what counts as “good health” seems reasonably clear, well-being remains a highly contested concept.^25^ ^,^^26^ ^,^^27^ There is little variation in what counts as good health across individuals, and good health is largely independent of individual aims and values. Conversely, the constituents of well-being vary significantly across people, and are highly dependent on individual aims and values.^28^ They also are evaluated holistically (i.e., well-being depends on the ways goods are combined, not simply on their number), and resist comparison across individuals.This makes well-being challenging to measure, and as a result, makes it difficult to measure the impact of health on well-being.

Moreover, the contribution of health to well-being is not easily separable from the contribution of other causes and constituents of well-being.^29^ The value of a particular health state and its impact on well-being depends on various factors in addition to health. For example, blindness may be less restrictive in an accessible urban environment than in a rural environment, but more restrictive in a literate society than one that is nonliterate. This makes it difficult to determine the (dis)value of blindness itself and its corresponding impact on well-being.

## “Recovery” in DOC

We can see these ambiguities in the relationship between health and well-being borne out in the ways that clinicians and families understand recovery in patients with prolonged DOC. From a clinical perspective, “recovery” generally refers to the recovery of specific target behaviors, eventually leading to functional independence. For example, the American Academy of Neurology Practice Guidelines use “recovery” primarily to denote recovery of consciousness, or improvement in degree of functional impairment.^30^ Assessment tools used to measure patient recovery after significant brain injury, such as the Glasgow Outcome Scale, Disability Rating Scale, and Coma Recovery Scale-Revised similarly reflect this focus on recovery of functional capacities, including command-following, object recognition and functional use, and reliable communication.^31^ Accordingly, from a clinical perspective, the decision to either continue or withdraw life-sustaining treatment is based largely on the expectation of whether or not the patient will regain sufficient physical and mental function, for example, to perform tasks of daily living, to live independently, or to live with some level of supportive care.

Conversely, surrogate decisionmakers and families often adopt a more general understanding of “recovery.” For them, recovery implies something like recovery to a quality of life the patient would find acceptable.^32^ ^,^^33^ Accordingly, treatment decisions are based on the possibility that the patient will eventually regain the kind of life they would have wanted for themselves, or what is in their “best interests” given the values they had preinjury.

The advantage of operationalizing recovery in terms of physical function is that it allows for objective assessment, using standardized and validated behavioral scales that are easy to administer. The disadvantage, however, is that it is unclear to what extent physical function influences well-being, or even functions as an indicator of well-being overall. Insofar as the aim of surrogate decisionmakers is to make treatment decisions on the basis of the patient’s possible future quality of life, a focus on recovery of physical function provides only a partial ground for decisionmaking.

## The Relationship Between Health and Well-Being

The claim that physical function has a significant impact on overall health should be uncontroversial. Other things being equal, lacking the use of one’s legs makes one “less healthy” than one otherwise would have been. However, it may not be the case that this also makes one “less well-off” (i.e., have lower well-being overall). Indeed, there is considerable evidence of people reporting high levels of well-being in spite of chronic illness or disability.^34^ ^,^^35^ ^,^^36^ ^,^^37^ This suggests that one cannot assume a particular change in well-being, based on a particular change in health state.

One way that the objective aspects of health and well-being can diverge is exemplified by the “disability paradox”: the tendency of patients with significant disability or chronic illness to report higher levels of subjective well-being or quality of life than would be predicted by others, based on objective factors such as their level of physical function.^38^ ^,^^39^ The fact that healthy individuals tend to systematically evaluate the well-being of individuals with disabilities or chronic illnesses as poor suggests that they judge these health states as having a negative impact on well-being. Conversely, the fact that people report high levels of well-being despite chronic illness or disability suggests that their health state has only a limited impact on their well-being, and thus, their quality of life overall.

One way of accounting for the disability paradox is by claiming that people with chronic illness or disabilities are not reliable sources of information about their own well-being. In the process of adapting to a new health state, people may deny the realities of their condition, or simply lack insight into the ways it affects their life. They may also adopt new standards by which they judge their own health or well-being, a phenomena known as “response shift.”^40^ ^,^^41^ ^,^^42^ This could lead to those with severe illness or disability over-estimating their “true” well-being.

Conversely, the disability paradox can also be explained by the fact that healthy people tend to overestimate the emotional impact that chronic illness or disability will have on their lives. This can result from a “focusing illusion”: the tendency to exaggerate the importance of any single factor when evaluating overall quality of life.^43^ When considering the quality of life of those with chronic illness or disability, healthy people may focus on the most obvious changes in circumstances disability causes, or only on those domains of life likely to be most affected by disability, and thus potentially overestimate the impact on their well-being overall. Similarly, healthy people may have difficulty imagining how they might adapt to their circumstances, and thus arrive at overly pessimistic predictions about the impact of chronic illness or disability.^44^

Even if we grant that the self-reports of individuals with chronic illness or disability are not completely reliable measures of their own well-being, it is difficult to deny that a central part of the impact of health on well-being is based on how it is experienced by the patient. That is, the impact of health on well-being must be judged, at least in part, “from the inside.” In highly unfamiliar circumstances, such as being in a prolonged DOC, this sort of evaluation is likely to be very difficult for healthy individuals (including surrogate decisionmakers), and may lead to estimates of well-being that are not well-aligned with those of the patient. Indeed, focusing primarily on recovery of physical function is likely to give at best a partial assessment of the well-being of these patients, because the ways in which a health state affects physical function is only one of many ways in which health can affect well-being. Moreover, these effects may not be fully appreciated “from the outside,” and require some insight into how these health states are actually experienced by the individual with prolonged DOC.

Appreciation of this problem has led to the development of specialized quality of life assessments for patients with severe brain injury.^45^ ^,^^46^ These kinds of assessments are designed to capture aspects of patient well-being that generic assessments of functional recovery may not, and thus, are more attuned to the consequences of severe brain injury on patient quality of life. By focusing on those domains of life which patients themselves report as being most relevant to their quality of life, these assessments can generate a more accurate picture of how the patient is actually faring by their own standards, as opposed to whether or not they satisfy certain objective criteria that may be less important to their overall quality of life.

However, judgements based on these assessments necessarily make an inference about the relative importance of each domain to overall quality of life. For example, a health-related quality of life assessment may ask a participant (or a proxy responding on their behalf) to evaluate their degree of social connectedness with others. If the patient rates this domain as poor, one might infer that their overall quality of life is thereby diminished. However, if the participant does not value social connectedness, the poor quality of their social connectedness may not affect their well-being, and thus, ought to receive less weight in determining their quality of life overall. In this example, the quality of life assessment may provide an inaccurate account of the individual’s quality of life, because it fails to evaluate quality of life with more weight on domains relevant to the specific participant. This is a problem for any assessment of overall quality of life based on domain-specific factors, unless the relative importance of each domain can also be determined.

Whether because of uncertainties about the relationship between physical health and well-being, or because of the limitations of evaluating well-being “from the outside,” there are serious problems with inferring the well-being of patients with prolonged DOC based on the presence or absence of physical function. Furthermore, even if clinicians and surrogate decisionmakers did have a clear sense of how a patient’s physical function impacted their well-being, it is a further question to determine how the patient would be faring overall. There is clearly more to how our life is going for us than our health state. Indeed, this seems to be what is at issue, at least for many families, when evaluating patient recovery and making decisions about further treatment and care. Even if we knew the extent to which the health state of a patient with prolonged DOC impacted their well-being, we still may not have a clear sense of their quality of life overall. It may also be the case that there is more to our overall quality of life than our well-being. That is, there may be things that we think are valuable contributors to a “good life,” but not because they make us better or worse off. For example, I might think that it is important to live my life in a morally upstanding way, but I need not think that doing so would make me better off.

Accordingly, we argue that when discussing the patient’s prognosis with surrogate decisionmakers, clinicians ought to clearly express the context in which prognosis is being judged. Specifically, recovery (or expectations for recovery) should be described in terms of awareness, functional status, and other dimensions potentially related to quality of life, rather than recovery ‘overall’. It should be acknowledged that quality of life is not reducible to physical function, and although changes in awareness or other functions may impact it significantly, their relationship to prospective quality of life will likely differ for individual patients. For example, several commentators have claimed that patients with CMD are actually worse-off than patients with VS/UWS, because the presence of consciousness, in the absence of other capacities (e.g., for communication), allows for greater suffering than if the patient remained unconscious.^47^ ^,^^48^ Increased awareness, such as would occur in a patient progressing from coma or VS/UWS to CMD or MCS, for example, may simply mean the patient is more aware of how poor their quality of life is. Equally, however, deficits in a particular domain should not be taken to imply a poor quality of life overall. Patients with prolonged DOC may retain sufficient cognitive capacities to experience pleasure and enjoyment, as well as other factors which may contribute to quality of life. The central question is whether their quality of life is sufficiently high in the relevant domains that they have an acceptable quality of life based on their own standards. The answer, as aforementioned, cannot be gleaned directly from an accounting of physical function. If judgements of future quality of life incorporate factors such as the potential for positive affective experience (pleasure and the avoidance of pain), social interaction including relations with others and perceived social support, the ability to communicate, and emotional well-being, in addition to physical and cognitive function, our appreciation of it will be much more nuanced and patient-centered.^49^

## Evaluative and Experienced Well-Being in DOC

We have argued that the focus of prognostication in patients with DOC should shift away from recovery of function, to a more holistic conception of quality of life, focusing on patient well-being. In this respect, we are in agreement with recent changes to national guidelines in the UK.^50^ This shift in focus not only raises challenges in assessment of the sort discussed above, however, but also highlights important conceptual features of the prevailing understanding of quality of life, which have not been recognized in the context of patients with DOC.

Subjective well-being—how well a person is faring against their own standards and expectations—is a critical component of overall quality of life.^51^ Indeed, the belief that a patient with a prolonged DOC “would not want to live like this,” is a judgement about their subjective well-being. Research suggests that well-being can be understood as consisting of two components, what Daniel Kahneman and Jason Riis refer to as “experienced well-being” and “evaluated well-being.”^52^ Experienced well-being is based on the affective character of a particular moment in time, the answer to the question “how good or bad is your experience *now*?” Evaluated well-being is based on our memories and subsequent assessment of those experiences. Although they are distinct components of well-being, they can influence one another. For example, subjective evaluations of our life-experience (evaluated well-being) will be influenced by our emotional experiences at a given time (experienced well-being); a person that has recently experienced mostly bad moods is unlikely to be very satisfied with their life. Similarly, subjective evaluations of our life can also influence our affective state (e.g., thinking about how well my life is going can increase my mood).

Evaluated well-being and experiential well-being can also diverge. One reason for this is the role of memory in shaping our evaluation of a particular experience. For example, Kahneman and colleagues exposed participants to three trials, in which they would submerge their hands in painfully cold water. The first “short” trial lasted 60 seconds in 14°C water, whereas the second “long” trial lasted 60 seconds in 14°C, with an additional 30 seconds in water warmed to 15°C. The participants were then asked to choose which trial they would repeat. A significant majority chose to repeat the “long” trial, despite the fact that it involved a greater amount of a painful experience. Because our evaluations of an experience often focus on the worst or final moments (the so-called “peak-end rule”), the longer trial was *remembered* as being less painful, and formed the basis of the participant’s choice to repeat it.^53^ Other research has shown that reports of evaluated well-being can be influenced by the ordering of questions,^54^ and even the weather on the day of the report.^55^

Global ratings of life satisfaction—“how satisfied are you with your life as a whole”—are measures of evaluated well-being. But given the potential effects of memory on our evaluations, evaluated well-being may not give us an accurate indication of experienced well-being. This may leave us with a distorted picture of well-being overall.

The possibility of divergence between experienced and evaluated well-being has important implications for surrogate decisionmaking in patients with DOC. As mentioned above, surrogate decisionmakers generally understand recovery in terms of a life the patient would find worth living. Moreover, surrogates often incorporate their own perspective into an assessment of the patient’s quality of life (“I would not want to live like that”). This suggests that for surrogate decisionmakers, the future quality of life of the patient—on which treatment decisions are based—is understood in terms of evaluated well-being.

On the one hand, this seems justified. In the UK, surrogate decisionmaking on behalf of patients with DOC is governed by the Mental Capacity Act 2005.^56^ It requires that decisions on behalf of the patient be made in their best interests, and consider the wishes, feelings, beliefs, and values—past and present—that would be likely to influence their decision if they had the capacity to decide for themselves. Similarly, Ireland’s Assisted Decision-Making Capacity Act 2015 requires surrogate decisionmakers to give effect to the past and present will and preferences of the relevant person, and take into account their beliefs, values, and any other factors which the relevant person would likely consider for themselves.^57^ Recent UK court decisions suggest that the crucial question with respect to treatment decisions, including the withdrawal of artificial nutrition and hydration, is whether the patient will recover a quality of life they themselves would value.^58^ ^,^^59^

If the aim of surrogate decisionmaking is to reconstruct the patient’s evaluation of their prospective life—as suggested by these guidelines—focusing on evaluated well-being seems appropriate, given that evaluated well-being plays a crucial role in decisionmaking.

However, focusing on evaluated well-being at the expense of experienced well-being risks underestimating overall quality of life in patients that remain capable of positive affective experience. There are two related issues here. The first is that surrogate decisionmakers might err in judging how the patient would evaluate their life in a severely disabled state, because the standards by which they evaluate their life might have changed postinjury. The patient’s previously expressed values and wishes may no longer reflect how they would evaluate their life in the present.^60^ Yet even if surrogate decisionmakers are drawing on values and wishes that accurately represent the patient’s evaluated well-being, they might still be neglecting the patient’s experienced well-being. This is the second issue. If a patient’s moment-to-moment experiences are broadly positive, it seems reasonable to judge that they have at least an acceptable quality of life, even if their overall satisfaction with life is low. There is evidence to suggest that patients with CMD, as well as MCS, remain capable of physical pleasure,^61^ and may also retain sufficient cognitive function for enjoyable experience.^62^ With the proper treatment and care, these patients may attain a life that they experience as broadly positive moment-to-moment, even in the absence of significant functional recovery.

There are some practical advantages to focusing on the experienced component of well-being when trying to discern or predict the future quality of life of a patient with DOC, particularly in patients that are noncommunicative. Whereas the evaluated component of well-being depends on self-report and memory, aspects of experienced well-being (e.g., pleasure and pain) may be easier to assess using behavioral measures. There is also some evidence to suggest that experienced well-being can be assessed using neurophysiological measures.^63^ Furthermore, in patients that can communicate, reporting on their immediate experienced well-being may be less cognitively demanding than reporting on evaluated well-being.

There are also some limitations to constructing a patient’s overall well-being on the basis of their experienced well-being. Aggregating a series of positive or negative experiences to generate a picture of experienced well-being overall requires some means of weighing these experiences. For example, should a prolonged, mildly pleasant experience be given equal weight to a brief but highly unpleasant experience? Thus, this strategy may only provide a coarse-grained picture of experienced well-being of patients with prolonged DOC. Nevertheless, as an account of the subjective experience of patients with prolonged DOC, it should certainly factor into decisionmaking on their behalf.

We are not arguing that surrogate decisionmakers should focus on the experienced well-being of patients with DOC at the exclusion of evaluated well-being. How people evaluate their experience is an important component of their subjective well-being, and their overall quality of life. We would suggest, however, that concern with the overall picture of one’s life implied by evaluated well-being may be overstated in patients with prolonged DOC. Although there are limited empirical data in this regard with respect to patients with prolonged DOC, patients with locked-in syndrome may offer some insight.

Locked-in patients are incapable of voluntary movement (except, in most cases, for vertical eye movement), or verbal communication, but remain cognitively intact. Reports of locked-in patients suggest that the simple experiences of day-to-day life are the source of much of their frustration, as well as much of their enjoyment.^64^ Although these reports are themselves evaluative, they suggest that locked-in patients are less concerned with how their disability has impacted the overall narrative of their lives, and more focused on overcoming the immediate struggles they face or enjoying the pleasures that are available to them. In other words, they seem more concerned with the affective character of their daily experiences, than with overall life-satisfaction. Although covertly aware patients might experience their disability differently than locked-in patients experience theirs, it is plausible that their experienced well-being might be more representative of their overall quality of life than their evaluated well-being.

## Conclusion

Diagnosis and prognosis of patients with DOC remains challenging, and places a significant burden on surrogate decisionmakers. Advances in our understanding of these disorders over the last decade represent a significant step forward, and are beginning to be reflected in practice. Yet these advances can be undermined by discrepancies in the goals of care, brought on by differing perspectives of patient recovery. We have highlighted two possible ways in which these perspectives can diverge: inferring quality of life from functional recovery, and failing to account for experienced well-being in addition to evaluative well-being. Although attending to these facets of well-being and quality of life may make treatment decisions even more difficult for surrogate decisionmakers, they are necessary to fully appreciating the complexity of these patients.

## Notes

1.
Katz DI, Polyak M, Coughlan D, Nichols M, Roche A. Natural history of recovery from brain injury after prolonged disorders of consciousness: Outcome of patients admitted to inpatient rehabilitation with 1–4 year follow-up. Progress in Brain Research 2009;177:73–88.1981889610.1016/S0079-6123(09)17707-5

2.
Whyte J, Nakase-Richardson R, Hammond FM, McNamee S, Giacino JT, Kalmar K, et al. Functional outcomes in traumatic disorders of consciousness: 5-year outcomes from the National Institute on Disability and Rehabilitation Research Traumatic Brain Injury Model Systems. Archives of Physical Medicine and Rehabilitation 2013;94(10):1855–60.2373216410.1016/j.apmr.2012.10.041

3.
Giacino JT, Sherer M, Christoforou A, Maurer-Karattup P, Hammond FM, Long D, et al. Behavioural recovery and early decision making in patients with prolonged disturbance in consciousness after traumatic brain injury. Journal of Neurotrauma 2020;37(2):357–65.3150249810.1089/neu.2019.6429PMC6964809

4.
Olaya J, Noe E, Navarro MD, O’Valle M, Colomer C, Moliner B, et al. When, how, and to what extent are individuals with unresponsive wakefulness syndrome able to progress? Functional independence. Brain Sciences 2020;10(12):990.10.3390/brainsci10120990PMC776566933339138

5.
Rodrigue C, Riopelle RJ, Bernat JL, Racine E. Perspectives and experience of health-care professionals on diagnosis, prognosis, and end-of-life decision making in patients with disorders of consciousness. Neuroethics 2013;6:25–36.

6.
Royal College of Physicians. Prolonged Disorders of Consciousness Following Sudden Onset Brain Injury: National Clinical Guidelines. London: RCP; 2020.

7.
Bruno MA, Vanhaudenhuyse A, Thibaut A, Moonen G, Laureys S. From unresponsive wakefulness to minimally conscious PLUS and functional locked-in syndrome: Recent advances in our understanding of disorders of consciousness. Journal of Neurology 2011;258:1373–84.2167419710.1007/s00415-011-6114-x

8.
Schiff ND. Cognitive motor dissociation following severe brain injuries. JAMA Neurology 2015;72(12):1413–5.2650234810.1001/jamaneurol.2015.2899

9.
See note 1, Katz et al., 2009.

10.
Estraneo A, Moretta P, Loreto V, Lanzillo B, Santoro L, Trojano L. Late recovery after traumatic, anoxic, or hemorrhagic long-lasting vegetative state. Neurology 2010;75(3):239–45.2055494110.1212/WNL.0b013e3181e8e8cc

11.
Nakase-Richardson R, Whyte J, Giacino JT, Pavawalla S, Barnett SD, Yablon SA, et al. Longitudinal outcome of patients with disordered consciousness in the NIDRR TBI Model Systems Program. Journal of Neurotrauma 2012;29(1):59–65.2166354410.1089/neu.2011.1829

12.
Giacino JT, Katz DI, Schiff ND, Whyte J, Ashman EJ, Ashwal S, et al. Practice guideline update recommendations summary: Disorders of consciousness. Neurology 2018;91(10):450–60.3008961810.1212/WNL.0000000000005926PMC6139814

13.
Schnakers C, Vanhaudenhuyse A, Giacino J, Ventura M, Boly M, Majerus S, et al. Diagnostic accuracy of the vegetative and minimally conscious state: Clinical consensus versus standardized behavioural assessment. BMC Neurology 2009;9:35.1962213810.1186/1471-2377-9-35PMC2718857

14.
Edlow BL, Claassen J, Schiff ND, Greer DM. Recovery from disorders of consciousness: Mechanisms, prognosis, and emerging therapies. Nature Reviews Neurology 2020;14:1–22.10.1038/s41582-020-00428-xPMC773461633318675

15.
See note 3, Giacino et al. 2020.

16.
Roozenbeek B, Lingsma HF, Lecky FE, Lu J, Weir J, Butcher I, et al. Prediction of outcome after moderate and severe traumatic brain injury: External validation of the International Mission on Prognosis and Analysis of Clinical Trials (IMPACT) and Corticoid Randomisation after Significant Head Injury (CRASH) prognostic models. Critical Care Medicine 2012;40(5):1609–17.2251113810.1097/CCM.0b013e31824519cePMC3335746

17.
Geurts M, Macleod MR, van Thiel GJMW, van Gijn J, Kappelle LJ, van der Worp BH. End of life decision in patients with severe acute brain injury. The Lancet Neurology 2014;13(5):515–24.2467504810.1016/S1474-4422(14)70030-4

18.
Leblanc G, Boutin A, Shemilt M, Lauzier F, Moore L, Potvin V, et al. Incidence and impact of withdrawal of life-sustaining therapies in clinical trials of severe traumatic brain injury: A systematic review. Clinical Trials 2018;15(4):398–412.2986589710.1177/1740774518771233

19.
Turgeon AF, Lauzier F, Simard JF, Scales DC, Burns KE, Moore L, et al. Mortality associated with withdrawal of life-sustaining therapy for patients with severe traumatic brain injury: A Canadian multicentre cohort study. Canadian Medical Association Journal 2011;183(14):1581–8.2187601410.1503/cmaj.101786PMC3185074

20.
Graham M. Burying our mistakes: Dealing with prognostic uncertainty after severe brain injury. Bioethics 2020;34(6):612–9.3212444810.1111/bioe.12737PMC7318633

21.
Kitzinger C, Kitzinger J. Court applications for withdrawal of artificial nutrition and hydration from patients in permanent vegetative state. Journal of Medical Ethics 2016;42:11–7.2648657110.1136/medethics-2015-102777PMC4717453

22.
Kuehlmeyer K, Palmour N, Riopelle R, Bernat JL, Jox RJ, Racine E. Physicians’ attitudes toward medical and ethical challenges for patients in the vegetative state: Comparing Canadian and German perspectives in a vignette survey. BMC Neurology 2014;14:119.2489832910.1186/1471-2377-14-119PMC4064260

23.
Huxtable R. Dying too soon or living too long? Withdrawing treatment from patients with prolonged disorders of consciousness after Re Y. BMC Medical Ethics 2019;20(1):91.3188495810.1186/s12910-019-0424-4PMC6936052

24.
Bognar G, Hirose I. The Ethics of Health Care Rationing: An Introduction. London: Routledge; 2014.

25.
Griffin J. Well-being: Its Meaning, Measurement, and Moral Importance. Oxford: Oxford University Press; 1986.

26.
Sumner LW. Welfare, Happiness, and Ethics. Oxford: Oxford University Press; 1996.

27.
Hausman DM. Valuing Health: Well-being, Freedom, and Suffering. Oxford: Oxford University Press; 2015.

28.
See note 27, Hausman, 2015.

29.
Broome J. Measuring the burden of disease by aggregating well-being. In Murray CJL, Salomon JA, Mathers CD, Lopez AD, eds. Summary Measures of Population Health. Geneva: World Health Organization; 2002.

30.
See note 12, Giacino et al., 2018.

31.
Nichol AD, Higgins AM, Gabbe BJ, Murray LJ, Cooper DJ, Cameron PA. Measuring functional and quality of life outcomes following major head injury: Common scales and checklists. Injury 2011;42(3):281–7.2114505910.1016/j.injury.2010.11.047

32.
Kitzinger C, Kitzinger J. Withdrawing artificial nutrition and hydration from minimally conscious and vegetative patients: Family perspectives. Journal of Medical Ethics 2015;41:157–60.2442575310.1136/medethics-2013-101799PMC4316914

33.
Jox RJ, Kuehlmeyer K, Klein AM, Herzog J, Schaupp M, Nowak DA, et al. Diagnosis and decision making for patients with disorders of consciousness: A survey among family members. Archives of Physical Medicine and Rehabilitation 2015;96(2):323–30.2544919210.1016/j.apmr.2014.09.030

34.
Mehnert T, Krauss HH, Nadler R, Boyd M. Correlates of life-satisfaction in those with disabling conditions. Rehabilitation Psychology 1990;35(1):3–17.

35.
Eisenberg MG, Saltz CC. Quality of life among aging spinal cord injured persons: Long term rehabilitation outcomes. Spinal Cord 1991;29:514–20.10.1038/sc.1991.741775357

36.
Gill CJ. Health professionals, disability, and assisted suicide. An examination of relevant empirical evidence and replay to Batavia. Psychology, Public Policy, and Law 2000;6(2):526–45.12661539

37.
Mailhan L, Azouvi P, Dazord A. Life satisfaction and disability after severe traumatic brain injury. Brain Injury 2005;19(4):227–38.1583286910.1080/02699050410001720149

38.
Albrecht GL, Devlieger PJ. The disability paradox: High quality of life against all odds. Social Science and Medicine 1999;48(8):977–88.1039003810.1016/s0277-9536(98)00411-0

39.
Amundson R. Quality of life, disability, and hedonic psychology. Journal of the Theory of Social Behaviour 2010;40(4):374–92.

40.
Sprangers MAG, Schwartz CE. Integrating response shift into health-related quality of life research: A theoretical model. Social Science and Medicine 1999;48(11):1507–15.1040025310.1016/s0277-9536(99)00045-3

41.
Menzel P, Dolan P, Richardson J, Olsen JA. The role of adaptation to disability and disease in health state valuation: A preliminary normative analysis. Social Science and Medicine 2002;55(12):2149–58.1240912810.1016/s0277-9536(01)00358-6

42.
Ubel PA, Loewenstein G, Schwarz N, Smith D. Misimagining the unimaginable: The disability paradox and health care decision-making. Health Psychology 2005;24(4):S57–62.1604542010.1037/0278-6133.24.4.S57

43.
Kahneman D, Krueger AB, Schkade D, Schwarz N. Would you be happier if you were richer? A focussing illusion. Science 2006;312(5782):1908–10.1680952810.1126/science.1129688

44.
See note 42, Ubel et al., 2005.

45.
von Steinbuchel N, Wilson L, Gibbons H, Hawthorne G, Hofer S, Schmidt S, et al. Quality of life after brain injury (QOLIBRI): Scale validity and correlates of quality of life. Journal of Neurotrauma 2010;27:1157–65.2021060210.1089/neu.2009.1077

46.
Tung J, Speechley KN, Gofton T, Gonzalez-Lara LE, Graham M, Naci L, et al. Towards the assessment of quality of life in patients with disorders of consciousness. Quality of Life Research 2020;29:1217–27.3183865510.1007/s11136-019-02390-8

47.
Kahane G, Savulescu J. Brain damage and the moral significance of consciousness. The Journal of Medicine and Philosophy 2009;34(1):6–26.1919369410.1093/jmp/jhn038PMC3242047

48.
Hawkins J. What is good for them? Best interests and severe disorders of consciousness. In Sinnott-Armstrong W, ed. Finding Consciousness: The Neuroscience, Ethics, and Law of Severe Brain Damage. Oxford: Oxford University Press; 2016.

49.
Graham M. A fate worse than death? The well-being of patients diagnosed as vegetative with covert awareness. Ethical Theory and Moral Practice 2017;20:1005–20.

50.
See note 6, Royal College of Physicians, 2020.

51.
Djikers M. Quality of life after traumatic brain injury: A review of research approaches and findings. Archives of Physical Medicine and Rehabilitation 2004;85(S2):21–35.10.1016/j.apmr.2003.08.11915083419

52.
Kahneman D, Riis J. Living, and thinking about it: Two perspectives on life. In Huppert FA, Baylis N, Keverne B, eds. The Science of Well-Being. Oxford: Oxford University Press; 2005.

53.
Kahneman D, Frederickson BL, Schreiber CA, Redelmeier DA. When more pain is preferred to less: Adding a better end. Psychological Science 1993;4(6):401–5.

54.
Strack F, Martin LL, Schwarz N. Priming and communication: Social determinants of information use in judgments of life satisfaction. European Journal of Social Psychology 1988;18(5):429–42.

55.
Connolly M. Some like it mild and not to wet: The influence of weather on subjective well-being. Journal of Happiness Studies 2013;14:457–73.

56.
Mental Capacity Act 2005 (c.9). London: The Stationary Office. Available at https://www.legislation.gov.uk/ukpga/2005/9/section/4.

57.
Assisted Decision-Making (Capacity) Act 2015 (No. 64 of 2015). Dublin: Houses of the Oireachtas. Available at http://www.irishstatutebook.ie/eli/2015/act/64/section/8/enacted/en/html.

58.
*Briggs v. Briggs* [2016] EWCOP 53.

59.
*NHS Trust v. Y* [2018] UKSC 46.

60.
Graham M. Precedent autonomy and surrogate decision-making after severe brain injury. Cambridge Quarterly of Healthcare Ethics 2020;29(4):511–26.3289277010.1017/S0963180120000286PMC7525111

61.
Graham M. Can they feel? The capacity for pain and pleasure in patients with cognitive motor dissociation. Neuroethics 2019;12(2):153–69.3198393110.1007/s12152-018-9361-zPMC6951816

62.
Naci L, Graham M, Owen AM, Weijer C. Covert narrative capacity: Mental life in patients thought to lack consciousness. Annals of Clinical and Translational Neurology 2017;4(1):61–70.2807831610.1002/acn3.376PMC5221458

63.
Davidson RJ. Well-being and affective style: Neural substrates and biobehavioural correlates. Philosophical Transactions of the Royal Society of London 2004;359:1395–411.1534753110.1098/rstb.2004.1510PMC1693421

64.
See note 60, Graham 2020.

